# Supplementary material for: Prevalence of anemia in predialysis chronic kidney disease: Is the study center a significant factor?
Source: PLoS One. 2020 Apr 2;15(4):e0230980. doi: 10.1371/journal.pone.0230980 (PMC7117725; doi:10.1371/journal.pone.0230980)
Supplement: S1 Table — (DOCX) [file pone.0230980.s001.docx]

**S1 Table. Baseline characteristics of the patients grouped according to the presence of severe anemia.**

|  | **Hg<10 g/dL**  **(n=133)** | **Hg≥10 g/dL**  **(n=838)** | **p-value** |
| --- | --- | --- | --- |
| Center  A  B  C | 77 (57.9)  29 (21.8)  27 (20.3) | 341 (40.7)  328 (39.1)  169 (20.2) | <0.001 |
| Age (years) | 65.5±14.5 | 65.2±13.7 | 0.638 |
| Gender (male), n (%) | 48 (36.1) | 415 (49.5) | 0.004 |
| DM, n (%) | 74 (55.6) | 384 (45.8) | 0.035 |
| Menopause, n (%) | 74 (87.1) | 376 (88.9) | 0.766 |
| Hgb (g/dL) | 9.2±0.7 | 12.3±1.5 | <0.001 |
| Htc (%) | 28.4±2.7 | 37.4±4.6 | <0.001 |
| MCV (fL) | 85.3±7.0 | 86.1±5.5 | 0.658 |
| Creatinine (mg/dL) | 3.0±1.6 | 2.0±1.1 | <0.001 |
| eGFR (mL/min/1.73m^2^) | 25.0±13.7 | 37.5±13.4 | <0.001 |
| eGFR  Stage 3 (30-59)  Stage 4 (15-29)  Stage 5 (<15) | 40 (30.1)  60 (45.1)  33 (24.8) | 593 (70.8)  194 (23.1)  51 (6.1) | <0.001 |
| CRP (mg/L) | 22.6±31.5 | 10.5±19.8 | <0.001 |
| iPTH (pg/mL) | 205.1±160.9 | 126.9±146.5 | <0.001 |
| Iron (µg/dL) | 55.8±32.6 | 66.1±28.1 | <0.001 |
| TIBC (µg/dL) | 246.3±100.7 | 288.6±75.4 | <0.001 |
| TSAT (%) | 0.29±0.31 | 0.25±0.18 | 0.443 |
| TSAT<20%, n (%) | 57 (44.9) | 322 (42.5) | 0.613 |
| Ferritin (ng/mL) | 239.9±302.6 | 119.3±165.0 | <0.001 |
| Ferritin <100 (ng/ml),  n (%) | 46 (36.2) | 498 (62.3) | <0.001 |
| Vitamin B12 (pg/mL) | 396.9±300.7 | 362.8±277.0 | 0.140 |
| Folate (ng/mL) | 8.4±5.7 | 8.6±5.1 | 0.206 |
| Vitamin B12 use, n (%) | 23 (17.3) | 150 (17.9) | 0,865 |
| Folate use, n (%) | 10 (7.5) | 58 (6.9) | 0.946 |
| Iron use, n (%) | 62 (46.6) | 190 (22.7) | <0.001 |
| ESA use, n (%) | 52 (39.1) | 55 (6.6) | <0.001 |

PHD and malignancy were excluded. Values are presented as mean±standard deviation for the continuous variables and frequency (percentage) for the categorical variables.

CRP: C-reactive protein, DM: diabetes mellitus, eGFR: estimated glomerular filtration rate, ESA: erythropoiesis stimulating agent, Hgb: hemoglobin, Htc: hematocrit, iPTH: intact parathyroid hormone, MCV: mean corpuscular volume, PHD: primary hematological disease, TIBC: total iron binding capacity, TSAT: transferrin saturation ratio.
