# Supplementary material for: Prevalence of anemia in predialysis chronic kidney disease: Is the study center a significant factor?
Source: PLoS One. 2020 Apr 2;15(4):e0230980. doi: 10.1371/journal.pone.0230980 (PMC7117725; doi:10.1371/journal.pone.0230980)
Supplement: Attachment — Submitted filename: Plos 1 , Reviewer 2020-1, Anemia among Pre CKD patients.docx [file pone.0230980.s002.docx]

Plos 1

1. [ABSTRACT];	Title and even apart of objective indicated that centre and or centres based treatment / services will affect the treatment, but abstract results didn’t indicated anything like that. I will suggest to incorporate related findings, if possible. Otherwise, rephrase title or objective part………………………………………………………………………………………………………………..
2. [INTRODUCTION]; Introduction last sentence is different than title and even with abstract objective…………………………………………………………………………………………………………………………...
3. This section is not sufficient to provide enough background of the objectives, it is suggested to elaborate……………………………………………………………………………………………………………………….
4. What was the inclusion criteria for this current project………………………………………………..…….
5. To determine the incidence by getting this 6-7 months data from THREE centres are sufficient..? Shall we generalized this Incidence at country level…?........................................
6. Baseline data included: sex, age, causes of kidney disease, presence of menopause, diabetes mellitus, primary hematologic disease, and malignancy… Why only these factors, how about hypertension?.............................................................................................................................
7. Provide reference for the statement of “Anemia was defined by hemoglobin (Hgb) level below 12g/dL and severe anemia was defined by Hgb level below 10g/dl”………………………….
8. MDRD formula is practicing in the centre or any other reason to select this formula……………
9. Table 1; It would be looking more better than existing , if author convert this into descriptive statistics, as three centres comparison might not be that much fruitful sound. But, Still authors like to keep as it is then they have to provide all three centres background. I mean all three centres are associated with tertiary level referral hospitals and how much bed size and CKD population are there. Further, where these centres are located, and the general population….?.......................................................................................................................
10. It is suggested to avoid abbreviations in the heading/sub-heading, like “ Anemia Parameters and the use of **ESA** and Iron”………………………………………………………………………………..
11. Table 2; P supposed to be p-value…………………………………………………………………………
12. Conclusion should be more specific based on study findings………………………………..
